# Influence of soil heterogeneity on soybean plant development and crop yield evaluated using time-series of UAV and ground-based geophysical imagery

**DOI:** 10.1038/s41598-021-86480-z

**Published:** 2021-03-29

**Authors:** Nicola Falco, Haruko M. Wainwright, Baptiste Dafflon, Craig Ulrich, Florian Soom, John E. Peterson, James Bentley Brown, Karl B. Schaettle, Malcolm Williamson, Jackson D. Cothren, Richard G. Ham, Jay A. McEntire, Susan S. Hubbard

**Affiliations:** 1 Climate and Ecosystem Sciences Division, Lawrence Berkeley National Laboratory, 1 Cyclotron Road, Berkeley, CA 94720-8126 USA; 2 Environmental Genomics and Systems Biology, Lawrence Berkeley National Laboratory, 1 Cyclotron Road, Berkeley, CA 94720-8126 USA; 3 Chemical and Biomolecular Engineering, University of California, 201 Gilman Hall, Berkeley, CA 94720-1462 USA; 4 Department of Geosciences and Center for Advanced Spatial Technologies (CAST), University of Arkansas, Fayetteville, AR 72701 USA; 5 Department of Industrial Engineering, 4207 Bell Engineering Center, University of Arkansas, Fayetteville, AR 72701 USA; 6 Glennoe Farms LLC, Stuttgart, AR 72160 USA

**Keywords:** Imaging techniques, Geophysics, Environmental impact, Agroecology, Imaging and sensing

## Abstract

Understanding the interactions among agricultural processes, soil, and plants is necessary for optimizing crop yield and productivity. This study focuses on developing effective monitoring and analysis methodologies that estimate key soil and plant properties. These methodologies include data acquisition and processing approaches that use unmanned aerial vehicles (UAVs) and surface geophysical techniques. In particular, we applied these approaches to a soybean farm in Arkansas to characterize the soil–plant coupled spatial and temporal heterogeneity, as well as to identify key environmental factors that influence plant growth and yield. UAV-based multitemporal acquisition of high-resolution RGB (red–green–blue) imagery and direct measurements were used to monitor plant height and photosynthetic activity. We present an algorithm that efficiently exploits the high-resolution UAV images to estimate plant spatial abundance and plant vigor throughout the growing season. Such plant characterization is extremely important for the identification of anomalous areas, providing easily interpretable information that can be used to guide near-real-time farming decisions. Additionally, high-resolution multitemporal surface geophysical measurements of apparent soil electrical conductivity were used to estimate the spatial heterogeneity of soil texture. By integrating the multiscale multitype soil and plant datasets, we identified the spatiotemporal co-variance between soil properties and plant development and yield. Our novel approach for early season monitoring of plant spatial abundance identified areas of low productivity controlled by soil clay content, while temporal analysis of geophysical data showed the impact of soil moisture and irrigation practice (controlled by topography) on plant dynamics. Our study demonstrates the effective coupling of UAV data products with geophysical data to extract critical information for farm management.

## Introduction

Precision agriculture has become an important strategy for improving crop micromanagement, maximizing productivity and optimizing resource allocation (i.e., smart use of irrigation, fertilizers, and pesticides)^1^. Part of the innovation in precision agriculture is the use of sensing technologies such as remote sensing (satellite, UAV imaging), geophysics, and sensor networks. These technologies provide complementary information on environmental variables related to both plants and soil, through both direct and indirect measurements.

Remote sensing technologies have been extensively used in precision agriculture. In particular, the development of high-resolution satellite imagery systems (e.g., IKONOS (1999), QuickBird (2001), RapidEye (2008), WorldView (2007), and GeoEye (2008)) have provided resolutions ranging from about 2 to 5 m per pixel and improved revisiting time^2,3^. Remote sensing data have been used to evaluate various agriculture practices, including (among others) water management in irrigated agriculture^4^, fertilizers and fungicide applications and their economic impact^5^, site-specific nitrogen fertilizer management^6^, and yield estimation for various crop types. However, the spatial resolution is still a limiting factor for studies that require information at plant/canopy scale. Weather conditions (in particular, cloud coverage) are also a critical impediment to satellite image acquisitions.

Recently, unmanned aerial vehicles (UAVs) have become readily available for agricultural applications^7,8^. Compared to both satellite and airborne platforms, the UAVs’ higher spatial–temporal resolution enhances our capability to study plant phenology in more detail, from leaf to crop scale^9^. Furthermore, the continuous introduction of UAV-specific sensors (e.g., multispectral and hyperspectral cameras, LiDAR sensors, thermal cameras) and their easy deployment provides the necessary planning flexibility to better characterize plant dynamics through the whole growing period and to avoid unfavorable weather conditions^10^. Among the different applications, UAV systems have been successfully used to monitor photosynthetic activities^11^, assess water stress and nutrient deficits^8,12^, estimate crop biomass^13^, canopy structures^14^, as well as identify crop diseases^15^. The ability to estimate plant-specific properties at the microscale (centimeter scale), such as plant counting and density^16–19^, would also promote the identification of potential heterogeneous spatiotemporal patterns within crop fields. Such information can be beneficial in detecting areas that have unusual responses (e.g., low plant density, low plant development) to local agricultural practices.

In parallel, geophysics has been used for agricultural applications to derive near-surface soil properties^20^. Geophysical methods, such as electrical resistivity tomography (ERT) and electromagnetic induction (EMI), have been widely used to extract near-surface and subsurface information as an alternative to expensive and time-consuming grid soil sampling^21,22^. Soil properties—such as soil texture and composition, organic carbon content, and soil moisture—influence nutrient availability and biogeochemical activity, which in turn affect plant development and productivity. The soil apparent electrical conductivity (EC_a_) measured by these systems is particularly sensitive to soil moisture, texture, and salinity, and therefore it can be used to estimate the spatial variability of the soil properties in heterogeneous environments^20,23^. For example, soil EC_a_ has been used to map soil salinity and investigate the negative impact of high-salinity soil conditions on the crop yield^24^. Soil EC_a_ has been used as a proxy both to estimate inorganic nitrogen^25^ and to build a spatial model for organic carbon distribution^26^. Soil characterization based on soil EC_a_ has been also used to delineate soil homogeneous zones for management purposes^27^. Plant traits, such as ground-estimated leaf area index (LAI) and plant height were shown to correlate with soil texture spatial varaibility^28^. It was also observed that such relationships vary, depending on the presence of dry or wet (year) conditions.

The joint use of remote sensing and geophysical data has been previously explored in natural ecosystems^21,22,29,30^. It has been observed, for example, that plant phenotyping has a close relationship with belowground properties, such as soil composition and microbiology.

Remote sensing vegetation indices (VIs), such as the normalized differential vegetation index (NDVI) and the green chromatic coordinates (GCC), are well-known and widely used tools to characterize and monitor the plant vigor, which refers to the health status of a plant in terms of greeness, photosintethic activity, biomass, and are found to exhibit a strong correlation to soil EC_a_^31^.

The combined use of geophysical measurements, LiDAR data, and vegetation indices allowed researchers to model complex relations between spatial patterns, biogeochemical activity, and carbon fluxes and to identify and characterize ice-wedge polygonal regions in an Arctic tundra ecosystem^22,29^. Strong correlations between spatial variability in soil EC_a_ and plant community spatial distribution estimated by high-resolution satellite images were observed in mountainous watershed ecosystems^30^ as well. The coupling of above- and belowground soil properties in agriculture ecosystems has been applied to better understand soil–plant interactions. A co-variability analysis between soil EC_a_ and satellite-derived LAI showed a general positive correlation following similar spatial patterns^32^. Recently, depth-specific soil EC_a_ and sun-induced fluorescent activity were jointly used to investigate soil–plant interactions^33^, showing that the impact of soil at different depths on plant dynamics can be captured by using multicoil data inversions. In both studies, results showed that the soil moisture in the deeper soil highly impacted plant performance. A recent study found that ground-surface electromagnetic data were useful for estimating the spatial distribution of Bordeaux vineyard soil classes, and that the soil classes were spatially correlated with grapevine vigor estimated using airborne-based normalized difference vegetation index (NDVI) data^34^.

Although there have been recent advances in UAV platforms and sensors, their potential has not been fully exploited for investigating soil–plant co-variability. While a few studies have proposed techniques to count plants^16,35^, low and medium resolution satellite-derived products are often used for plant phenology and physiology characterization (i.e., plant’s physical, structural, developmental, and physiological properties). However, there are few studies investigating early-season plant phenology and plant growth, and their relationship to soil properties. Early-season plant spatial abundance and plant vigor, which can be effectively estimated by high-resolution UAV image, are critical for identifying anomalous areas and for providing easily interpretable information for better decision making (e.g., replanting, reanalysing the irrigation system, etc.). In early season, plant coverage is expected to be spatially homogeneous, especially when the seeds are planted at an even distance. However, many factors can impact plant development from the emergence stage until the final reproductive stage of maturity, such as extreme local environmental conditions of soil moisture and temperature. Furthermore, it remains a challenge to integrate multiple sources of information to infer the properties of interest in agriculture that affect plant development. For example, to the authors’ knowledge, there are no studies identifying key controls on plant growth or investigating the influence of initial plant assessment on crop yield by integrating geophysics and remote sensing techniques. In addition, the temporal variability of aboveground-belowground properties represents another important aspect for investigating soil–plant relationships. Such relationships can be affected by the changes in soil conditions due to drought periods or heavy rains, and can be captured through time-lapse soil geophysical characterization.

In this work, we investigate the soil–plant interaction within an agricultural ecosystem—including crop yield—by taking advantage of high-resolution UAV data and spatially extensive geophysical data. We assess the impact of soil heterogeneity on the spatial–temporal variability of plant development and crop yield. For this purpose, we propose a novel framework that includes multi-source data integration of UAV-derived imaging and geophysical data. Our framework consists of three steps: (1) developing algorithms for high-resolution UAV images for mapping plant structural (height) and physiological information; (2) characterizing the soil–plant coupled spatial and temporal heterogeneity; and (3) identifying key controls on plant development and crop yield by investigating the co-variability between soil properties and UAV-derived products.

The particular emphasis is on estimating early-season plant signatures and geophysics-based soil characterization over the field. The proposed procedure estimates the plant spatial abundance by identifying the pixels associated with the plants and computing the ratio within a unit area. The discrimination between plants and soil allows us to improve the estimation of plant vigor by minimizing the background effect of soil. In this work, we used the GCC computed from a high-resolution RGB camera to estimate plant physiological dynamics and measured in situ high-resolution multi-depth soil EC_a_ to account for the soil spatial heterogeneity. We then investigate the co-variability among high-resolution UAV-derived products and soil EC_a_ to assess the impact of soil spatial heterogeneity on plant development. Geo-referenced yield maps are used to investigate the relationship between plant development and actual crop yield. To the authors’ knowledge, this is the first study to integrate UAV and geophysical technologies to characterize spatiotemporal variability in both soil and plants, and to quantify their relationships to crop yield.

## Materials and methods

We demonstrate our approach using an agricultural field under active commercial cultivation of soybeans located in the Arkansas Delta. For this study, we acquired time-series datasets of UAV and geophysical data during the 2018 growing season. This provided the opportunity to investigate the relationship between plant growth and spatially heterogeneous soil physical properties, as well as to analyse the temporal dynamics of such a relationship. All the methodology and data collection comply with relevant institutional and national legislation.

### Site description and crop management

**Figure 1:**
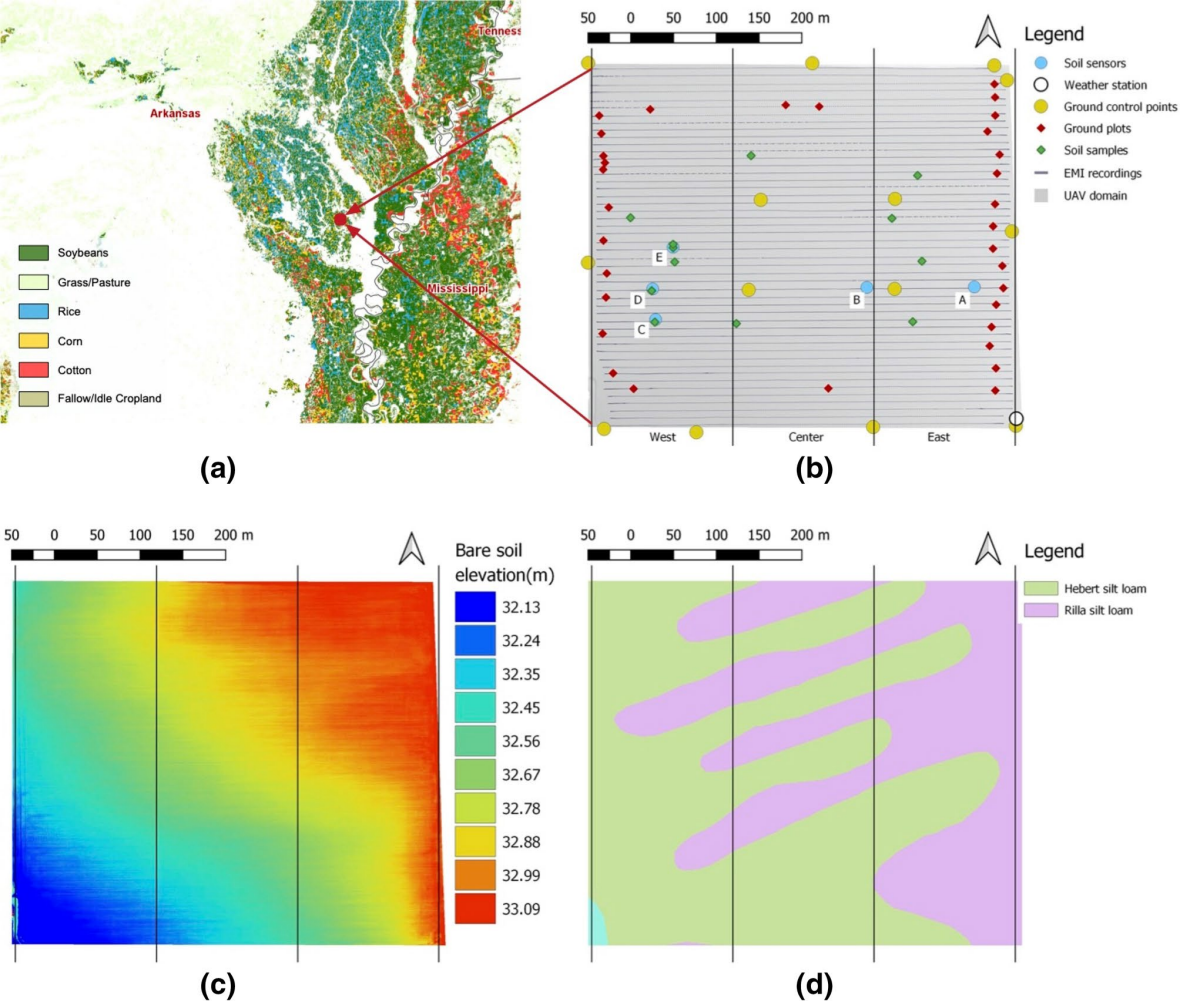
Study area located in Humphrey, Arkansas: (**a**) map produced by cropScape (USDA) showing part of the Delta region with the main agricultural land covers; (**b**) map of the field under study reporting the locations of the ground data collection; (**c**) map showing the topographic elevation derived by an UAV-based DEM, acquired acquisition prior to planting; (**d**) gSSurgo map provided by USDA, showing the soil types. Black vertical lines separate the east, center, and west sections used in the statistical analysis. Maps made with QGIS (v 3.6, https://www.qgis.org)^36^.

The investigated study area (34° 24.458′ N, 91° 40.462′ W), located in Humphrey, Arkansas, is a crop field of about 20 hectars that is under active commercial cultivation of soybeans (Fig. 1).

The study area is part of the Lower Mississippi River Basin’s (LMRB) alluvial plain^37^. This region is characterized by a wide range of cropland, such as soybean, corn, rice, grass, and pasture, supported by a humid subtropical climate with hot, humid summers and mild, slightly drier winters.

The crop field is adjacent to the oxbow Glenwood Lake (shown in Fig. 1) and is characterized by an alternating of Hebert silt-loam and Rilla silt-loam, according to the description provided by the gSSURGO map^38^. The Hebert profile consists of a very deep, poorly drained, moderately slowly permeable soil that formed in silty alluvium, while the Rilla profile consists of a very deep, well drained, moderately permeable soils that formed in reddish silty and loamy alluvium.

High-quality soybean seed requires three appropriate conditions for germination—soil moisture, temperature and oxygen. Provided that the soil is not saturated, oxygen concentration is not a limitation, but germination will not occur in flooded soil dueto lack of oxygen. Research shows that soybeans are in general very susceptible to soil saturation and anoxic conditions, which would provoke damage to the root system and in some case the termination of the plant growth^39^.

Planting started on April 21st in rows (raised beds) with a spacing of 0.9 m. Plants reached their first vegetative stage of emergence (VE) on the first days of May and developed their first node with fully developed unifoliate leaves around mid-May (V1). The stage of initial bloom, which represents the starting of the reproductive stage (R1–R8) after the last vegetative stage, started in mid-June, reaching the full maturity (R8) about the end of September. Subsequent to R8, the leaves dry then fall from the plant at the time of harvest, which was performed on October 24th.

A common channeled (furrowed) surface irrigation approach was used as an irrigation system, which consists of a plastic pipe (polytube) located along one side of the field to deliver water into the channels between the rows^40^. At the site, the plastic pipe was located on the east side of the field with the rows and furrows running east to west following the main slope gradient (Fig. 1) from east to west, in order to facilitate the water flow. Smoothing of the field’s ground surface was performed prior to the planting to ensure a good surface drainage. Irrigation started on June 12th, after the rain period, and performed every 14 days.

Temperature and precipitation were measured by a weather station that we installed in the southest corner of the field (Fig. 1). Based on the data collected, the average daily temperature increased from 14.6 to 24.7 °C between planting and the first vegetative stage (April–May), reached 27 °C during the vegetative period through the initial reproductive period (June–July), and decreased from 27 to 24 °C during the final period of maturity (August–September). The 2018 growing season was characterized by sporadic heavy-rain events, especially during the vegetative period (from April to mid-June), with a total cumulative precipitation (period April–September) of about 650 mm (Supplementary Figure S1). As a result of rain events, the soil moisture in April ranged from 0.17 (m^3^/m^3^) to 0.3 (m^3^/m^3^) (Supplementary Figure S2).

### Instrumentation, data acquisition, and preprocessing

#### UAV-data acquisition

Multitemporal image acquisitions were performed over the field using UAV-mounted sensors. The images were acquired on: May 8th, a few days after planting, May 28th, during the early vegetative development, and June 25th, July 23rd, September 19th, the beginning, middle, and end of the reproductive period.

We used the DJI Matrice 600 flying platform with the DJI FC350 on-board RGB (red, green, blue) camera as the primary image acquisition sensor. A flight plan to acquire high-resolution images at centimeter resolution was designed using this acquisition system. Flights were performed during the maximum Sun’s elevation and with clear sky to avoid possible changes in light conditions, at an altitude of about 60 m, with a 75% image overlap, reaching a ground resolution of 2 cm.

In order to minimize the impact of possible variations in illumination conditions during each acquisition, two normalized mosaics were computed by using the pictures of a reference panel taken before and after each flight. The final mosaic was obtained by averaging the two normalized mosaics.

A total of 13 Ground Control Points (GCP) were positioned along the field perimeter and within the field for the mosaic reconstruction (Fig. 1). These consisted of flat targets and were surveyed at each acquisition with a Topcon real‐time kinematic differential global positioning system (Hyper-V RTK-GPS). The system is composed of a rover and a fixed base station, ensuring high-resolution geolocation information at centimeter resolution. All the collected geolocation data were post-processed obtaining an average planar error of about 2 cm and a vertical error of about 3 cm.

We used the commercial PhotoScan (Agisoft) software for photogrammetry to construct the final mosaics. The software uses photogrammetry techniques based on structure from motion from multi-view stereo (SfM-MVS) images^42^. The processing produced mosaics with spatial resolution less than 10 cm, but the mosaics used in this work have a have a pixel size of 10 cm. Additionally, we used the software to compute digital elevation models (DEMs) from the RGB images with a final vertical resolution less than 5 cm.

#### Electromagnetic Induction (EMI) system

Soil EC_a_ was measured with an electromagnetic induction (EMI) system (CMD Mini-Explorer, GF Instruments), which consists of a transmitter and three receiver coils, allowing an effective acquisition of soil apparent electrical conductivity (EC_a_) averaged across a depth range of 0.5 m, 1.0 m, and 1.8 m from the surface. The instrument provides soil EC_a_ measurements in milliSiemes per meter (mS/m) and the in-phase in parts per thousand (not considered in this study). The system was mounted in a sled pulled by an all-terrain vehicle and supported by a Differential Global Positioning System (DGPS) in order to tag each measurement with high-resolution positioning information. Time-lapse soil EC_a_ data collection was performed four times, including before and during the growing season: April 21st, May 12th , June 11th, and July 9th. EMI acquisitons were planned according to the irrigation plan and UAV acquisitions. Since the EMI instrument is sensitive to changes in soil moisture, we avoided the collection during or right after irrigation events. The data were acquired consistently every 10 rows with a distance between traverses of about 9 m.

#### In situ soil monitoring, ground imagery, and soil sampling

Soil sensors (5TE, METER) were used for continuous acquisition of volumetric water content (VWC) and soil temperature. The sensors were deployed in five areas (A, B …, E, see Fig. 1) based on two factors: a) spatial varibaility of geophysical properties, b) differences in yield during the 2017 growing season (historical data available for this study). Each area has one logger that accomodates 4 sensors within a maximum radius of 4 m. To capture both shallower and deeper soil dynamics, we positioned 2 sensors vertically aligned at the depths of 12 cm and 25 cm on both the south (side1) and north (side2) of the logger. The areas A and D were positioned in high and low soil EC zones, respectively, while B was in an area of transition. Sensors A, B, and D were positioned along the same rows to forms two transects, one on the south and one on the north. Sensor E was positioned within a a very low 2017 yield in the west side of the field but at a mid-range of soil EC values. The logger C was positioned across a soil EC boundarie, but it stopped recording data at early season and was discarded from this analysis. The collected temprature data are used to correct the EMI acquisitions, whereas soil moisture is used to track the irrigation events and possible saturations. As a support system for the sensors’ data transmission, we used EM60G (METER) data loggers and ZENTRA cloud (METER) for cloud data storage.

Ground RGB images were taken at several plots (Fig. 1) for ground-based plant spatial abundance assessment. We used a wooden-frame of 1 m by 1 m to delimit the plot’s area and took a picture from above (1.4 m from the ground). This assessment will be used to validate UAV-based plant spatial abundance estimates.

Soil samples were collected at twelve locations (Fig. 1). Physical laboratory testing was performed at the Fayetteville Agriculture Diagnostic Laboratory of the University fo Arkanas to characterize soil properties, such as clay density, porosity, dry bulk density, volumetric water content, and organic matter. Such information was used for statistical analysis and to provide an interpretation of the soil EC_a_ measurements. No plant tissues or seeds were collected in this study.

#### Yield data from a combine harvester

Crop yield was harvested on October 24th. The combine harvester recorded yield information as cloud-point data in bushels per acre (bu/ac) and reported here in kilogram per hectare (kg/ha), and the relativre geolocation information. The combine harvester is able to collect 11 rows at once and records a point measurement every 2 s. Areas with artefacts were determined based on the swath parameter recorded during the harvesting. The wrong choice of the swath parameter on the combine harvester would produce unreliable yield values. Such areas were not considered in further analysis.

### Methodology

We designed a data analysis framework to combine the UAV optical images and soil geophysical measurements for characterizing plant phenological and physiological properties and soil spatial heterogeneity, respectively. We then performed statistical analysis of their co-variability to quantify the influence of soil properties on plant development. This framework is constructed by the following three steps:

1. UAV-based monitoring of plant dynamics: This step presents the data processing pipeline used for computing UAV products to characterize plant phenology and structure, such as plant spatial abundance, plant-specific vigor, and plant height.
2. EMI-based monitoring of soil characterization: This step focuses on quantifying the soil spatial variability. We perform statistical analyses to identify the relationship between soil EC_a_ signal and textural information derived by soil samples collected during the ground data acquisition.
3. Quantifying soil–plant spatiotemporal co-variability: This step focuses on the statistical analysis of plant physiological and structural properties and near-surface properties, as well as identifying key environmental factors.

#### UAV-based plant monitoring

**Figure 2:**
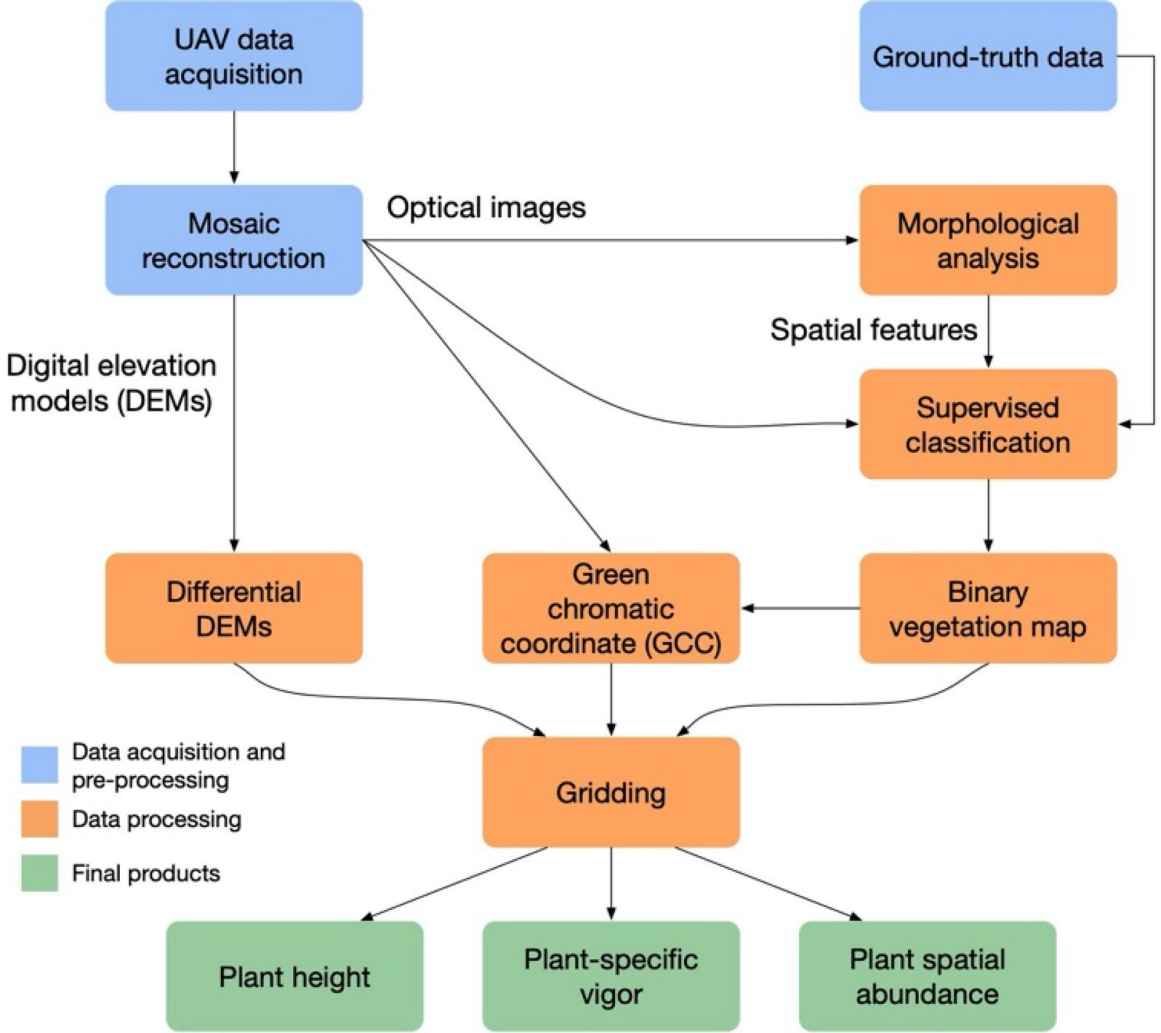
Pipeline developed for the UAV data processing.

Figure 2 depicts the data processing pipeline developed to assess the spatial variability of plant characteristics during the growing season. The steps to obtain the plant spatial abundance and plant vigor maps are presented in detail in the following sections.

##### Plant spatial abundance

Plant spatial abundance is estimated by the RGB mosaics. In the first step of the procedure, a vegetation map was obtained by performing a binary classification to discriminate the two classes of vegetation and soil. We used a supervised spectral-spatial image classification approach^43^ that combines both the spectral and the contextual (i.e., geometrical) information. The contextual information represents the pixel spatial arrangement and the spatial reletionship between adjacent pixels. By modelling such infromation, we can extract structures within the scene. For this step, we use mathematical operators, such as attibute profiles^44–46^ that belong to the image processing sub-field of mathematical morpohlogy. Such operators are 2D filters that act on regions of connected pixels based on the evaluation of an attribute (e.g., scale, defined as number of pixel composing the region). The advantage of using this operator is the ability to preserve the geometrical detail of the structures in the scene. In a filtered image (or feature), regions with similar properties (i.e., scale) are preserved or otherwise merged to their surroundings (i.e., filtered). A multiscale contextual characterization is obtained by applying a filter recursively and relaxing the scale parameter of the filter at each iteration, resulting in a stack of filtered images. An automatic procedure developed in^45^ is used to perform such a characterization. Such operation serves to extract homogeneous structures present in the scene at different spatial scales, allowing us to better delineate the soybean rows. The original RGB image, together with the filtered images, compose the feature space used as input to a support vector machine (SVM) algorithm with a radial basis function (RBF) kernel. The algorithm is based on the LIBSVM library^47^ developed for the MATLAB environment, using a one-against-one multiclass strategy. In a second step, we compute the spatial abundance of the detected plants as the percentage of pixels that belong to the vegetation class within a grid unit. In our study we chose a grid unit of 2 by 2 m, which would allow to obtain a good approximation of the local change, while capturing large scale patterns. The quality of the plant spatial abundance map is mainly dependent on the performance of the classification algorithm used to separate the plants from the soil in the background. To validate this product, we computed the classification performance based on ground-truth data and compared the UAV-based estimates of plant abundance to ground-based assessments. The ground assessment of the plant spatial abundance was performed by selecting 31 plots in conjunction of the UAV acquisition occurring in May 28th, 2018. The plots were located mainly along the east and west sides of the field within a distance that ranges between 12 and 25 m from the edge of the field towards the field center; and some plots were collected along the south and north sides within a distance of 50 m from the field edge. These plots were selected to captured representative spatial abundances, ranging from 0% (almost bare) to 60%. Ground images were systematically taken at a height of 1.4 m from the ground. From these images, we computed the GCC and identified a threshold to separate the plant covered area from the soil backgroun. The plant spatial abundance was then computed as the percentage of pixels identified as soybean over the entire area of the plot. All the ground-data were geolocated using the RTK-GPS system previously described.

##### Plant-specific vigor estimation

Remote-sensing-derived VIs have been extensively used as a proxy to estimate plant vigor and investigate plant physiology and response to possible ecosystem changes. Widely used VIs for plant characterization are in general derived by the near-infrared (NIR) channel (e.g., NDVI, SAVI, etc.), which is sensitive to changes in chlorophyll and leaf area. However, RGB-based indices have been developed and used in several phenology studies, showing their effectiveness in estimating plant characteristics compared to NIR-based VIs^48^. The GCC was chosen based on authors’ recent studies, showing that this RGB-based VI is less affected by saturation compared to NDVI^31^. GCC is formally defined as follows:

1 $${\text{GCC}} = \frac{{{\text{G}}_{{{\text{DN}}}} }}{{{\text{B}}_{{{\text{DN}}}} + {\text{G}}_{{{\text{DN}}}} + {\text{R}}_{{{\text{DN}}}} }},$$

where R, G, and B are the red, green, and blue channels, and DN refers to the pixel intensity values as digital numbers^41^. The plant-specific vigor was computed considering only those pixels identified as vegetation, the information for which is provided by the binary classification. In order to have products spatially comparable, the plant-specific vigor was transformed into a 2 by 2 m grid by computing the average within each grid unit. This procedure has the advantage to minimize the soil component in estimating plant vigor, avoiding the nonuniqueness of lower productivity versus increased soil coverage. Specifically, it allows a more accurate way of capturing the spatial variability of the plant’s vigor, in particular during the early vegetative stage, when the soil component is overabundant compared to the plant spatial abundance. This is a clear advantage over a satellite-derived VI with a coarser resolution that would provide an underestimation of plant vigor due to soil coverage.

##### Plant height estimation

Plant height was computed by subtracting the digital elevation model (DEM) of bare soil (i.e., reference DEM) from the digital surface model computed at each acquisition. The reference DEM was computed from acquisition occurred on May 8th, which was planned a week after planting so that it is not affected by surface disturbances by the planter. DEMs were computed using the PhotoScan software (Agisoft) during the mosaic reconstruction phase. We assume that the ground surface elevation stays the same during the growing season.

#### EMI-based soil characterization

We used the EC_a_ data to evaluate the spatial heterogeneity of soil properties and the temporal variability in soil moisture. We primarily used the EMI measurements associated with the 1 m effective depth, which is the depth range expected to encompass the soybean roots. Temperature corrections were applied to the point-cloud data for correcting the measured soil EC_a_ to the reference temperature of 25 °C. We employed the exponential model presented in Corwin and Lesch^49^ and developed by Sheets and Hendrickx^50^, and formally described as follows:

2 $${\text{E}}C_{25} = {\text{E}}C_{a} \cdot \left[ {0.4470 + 1.4034e^{{\left( { - T/26.815} \right)}} } \right],$$

where ${\text{E}}C\_\left\{ {25} \right\}$ represents the corrected soil EC_a_ at the reference temperature of 25 °C, ${\text{E}}C\_a$ represents the measured soil EC_a_, and ${\text{T}}$ represents the soil temperature at the time of the acquisition. The model was chosen based on the on the methodological comparison^51^, which shows that the exponential model provides very similar estimations compared to the widely used ratio model^31,52^ in the temperature range of 3–43 °C, but with a smaller total residual.

A spatial correction was applied to the data to resolve a spatial shift caused by the distance from the EMI instrument and the actual position of the GPS antenna, which were 4.5 m apart due to the system setup. We performed statistical analysis between EMI data and soil samples collected during the April data acquisition to identify the main control on the spatial variability of soil EC_a._

#### Co-variability among plant and soil signatures

We performed a suite of statistical analyses, based on the high-resolution data layers, on soil and plant characteristics (i.e., plant spatial abundance, plant vigor, plant height, soil EC_a_, and crop yield) to investigate the impact of soil properties on plant development during the growing season. For the analysis, we considered the yield point-cloud data as geo-reference.

Since the EMI and crop-yield data had a lower vertical resolution (along the y axis) of about 10 m (distance between rows selected for the data collection), we computed the average of each metric within a window of 20 m by 20 m. The exploratory data analysis included run charts and scatter plots to investigate the temporal evolution of plant development during the growing season, and the co-variability between plant characteristics and soil EC_a_. We evaluated the correlation between variables using Pearson’s correlation, as well as their spatial association. To control for the spatial non-independence, we derived a corrected Perason’s correlation by using the hypothesis testing procedure propsed by Clifford, Richardson, and Hémon (1989)^53^. The method uses Moran’s I^54^ to compute the spatial autocorrelation in the spatial data sets and estimate the correct degrees of freedom, which are then used to assess the significance of the correlation. The method uses the Sturges' formula to identify the number of distance classes^55^.

## Results

### UVA-derived products: plant spatial abundance, plant-specific vigor, and plant height

**Figure 3:**
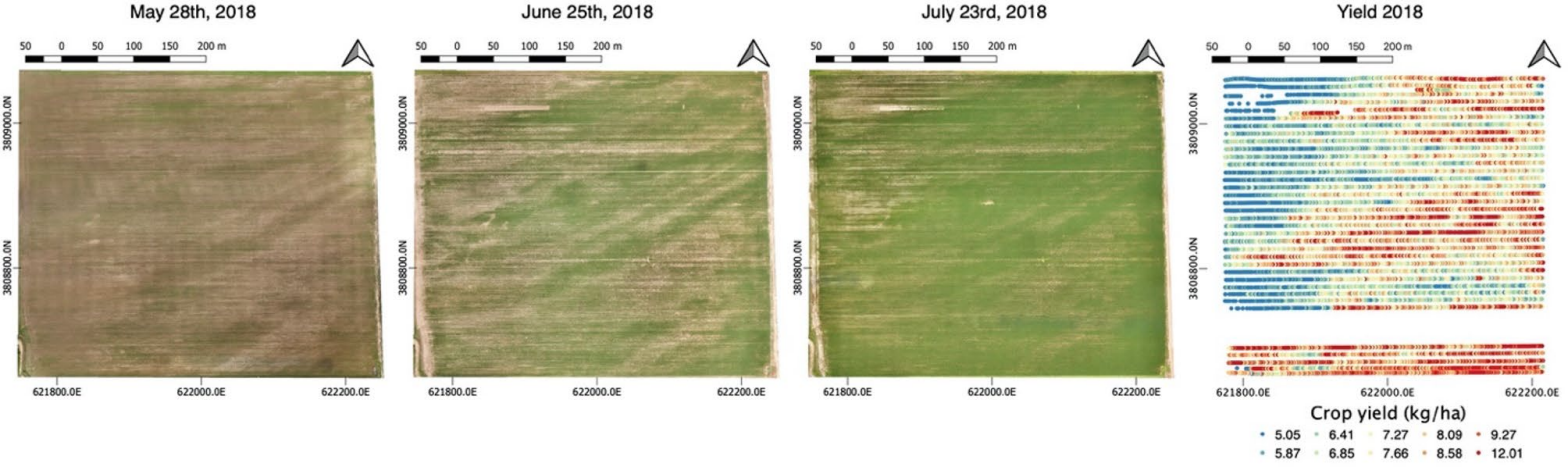
Time-lapse of UAV-RGB image acquired during the growing season (May 28th, June 25th, and July 23rd), and the point-cloud maps of the yield (kg/ha) acquired by the combine harvester (right figure). Maps made with QGIS (v 3.6, https://www.qgis.org)^36^.

Figure 3 shows the reconstructed multitemporal RGB mosaics at a spatial resolution of 10 cm, acquired over the field in May 28th, June 25th, and July 23rd, and the point-cloud yield map acquired by the combine harvester.

**Figure 4:**
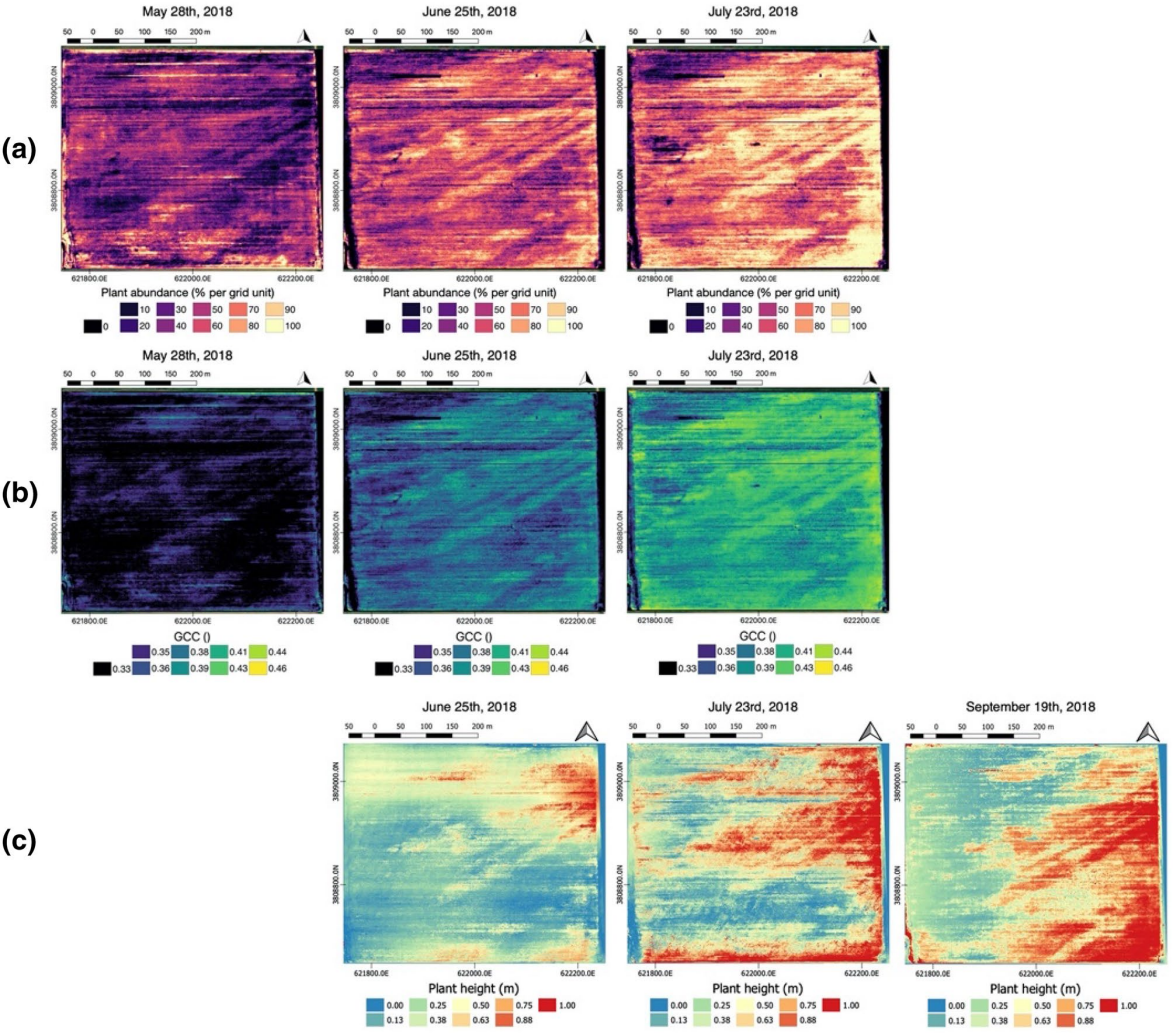
UAV products consisting of: (**a**) plant spatial abundance; (**b**) plant-specific vigor represented by the GCC index; and (**c**) plant height. The maps are vertically aligned accrding to their temporal acquisiton. Maps made with QGIS (v 3.6, https://www.qgis.org)^36^.

From each acquisition, DEMs were also extracted at the same pixel resolution. Plant spatial abundance, plant-specific vigor, and plant height (Fig. 4) were estimated from the RGB dataset shown in Fig. 3. All the computed products show a spatial heterogeneity in plant development over the growing season. Although plant spatial abundance and vigor generally increase over the growing season, the images clearly show persistent areas where plant spatial abundance and vigor remain low (depicted in dark colours, e.g., northwest, southeast). The products also identify areas where plant-specific vigor and plant spatial abundance degraded in the late part of the season; particularly in the west-center area of the field. Similarly, the plant height maps, which were computed for the acquisitions of June 25th, July 23rd, and September 19th, show how plant structure and development follow specific spatial patterns.

**Figure 5:**
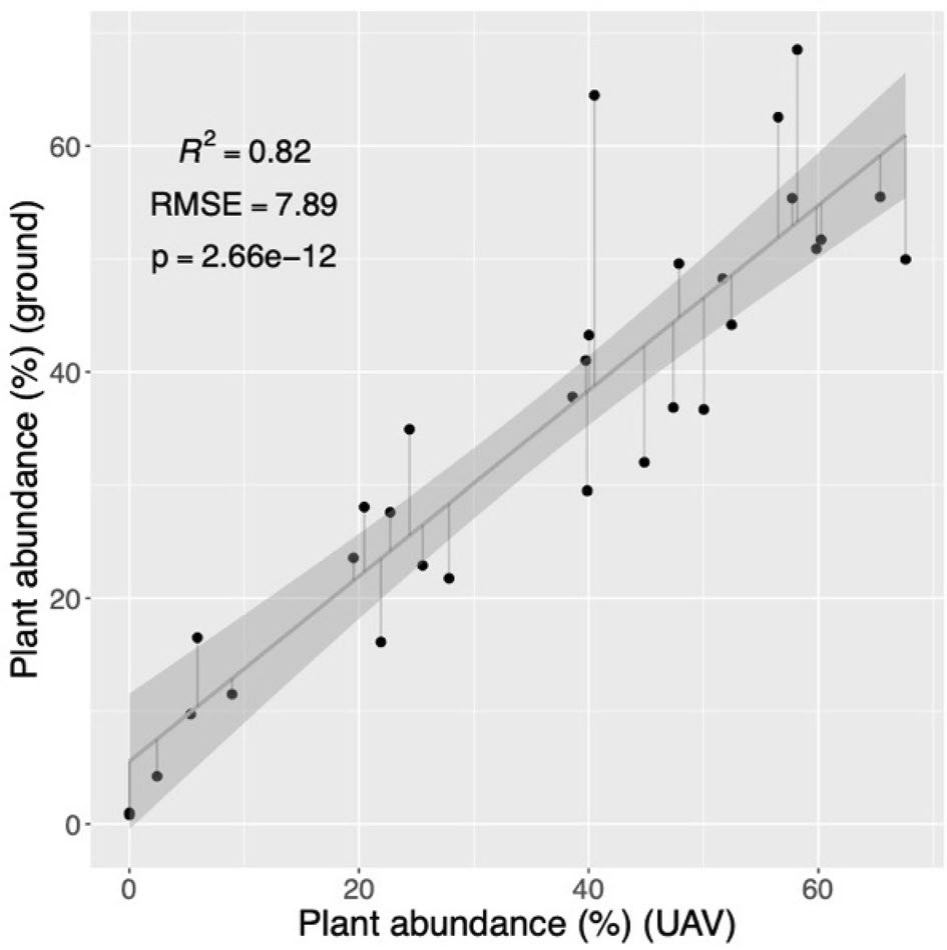
Scatterplot between ground and UAV-based plant spatial abundance. The analysis shows a high R^2^, indicating a strong agreement between the two estimations, which is also supported by a significant p-value. The scatterplot also shows the residuals, whose standard deviation is about 7.89 (RMSE).

**Figure 6:**
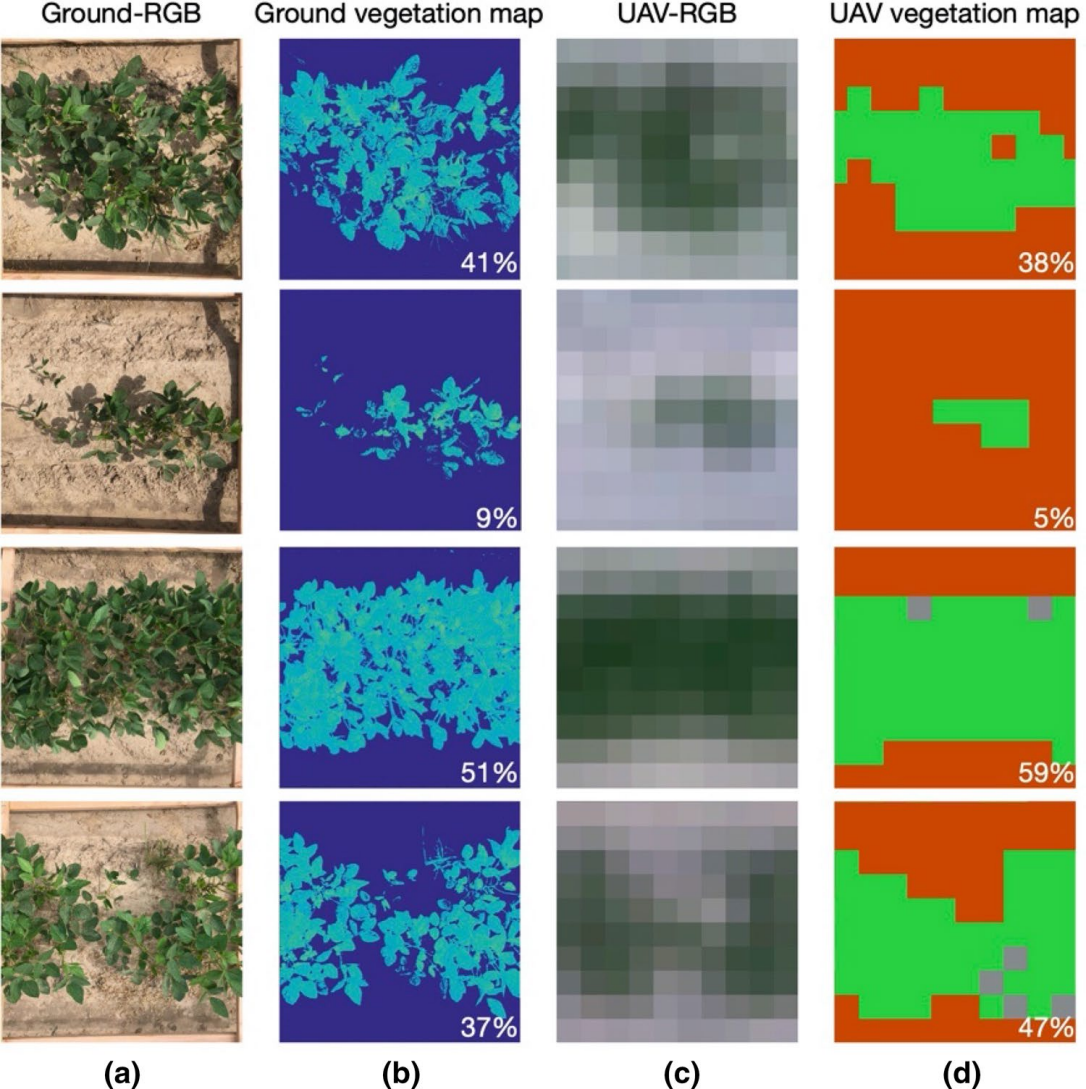
Ground assessment of the performance of the algorithm for extracting vegetation and computing the plant spatial abundance: (**a**) ground images of the plots captured by an RGB camera; (**b**) extraction of vegetated area for plant spatial abundance estimation; (**c**) UAV-RGB portion that covers the plot; (**d**) vegetation map obtained by the proposed method to estimate the plant spatial abundance, where green, red, and grey represent the vegetation, soil, and the unclassified classes, respectively. The estimated plant spatial abundance are reported for each case. Maps made with MATLAB R2017a (1994–2020 The MathWorks, Inc.)^56^.

The performance of the binary classification used to compute the plant spatial abundance and plant-specific vigor is assessed in terms of class accuracies, overall accuracy, and kappa coefficient averaged over fivefold. The metrics are reported in Table 1, together with the relative standard deviations. A quantitative assessment of the plant spatial abundance estimation was performed by investigating the correlation between the plant spatial abundace estimated via UAV and the one estimated based on ground observations. The validation analysis based on the 31 plots, depicted in Fig. 5, provided a R^2^ of 0.82, indicating that the UAV-based estimations of plant spatial abundance can explain most of the ground measurements’ variance. The significance of this result is supported by a p-value of 2.66 × 10^–12^. The figure also indicates the residual for each sample, whose standard deviation (root mean square error–RMSE) is about 7.89. A few examples of the ground estimation and relative UAV-based estimations are depicted in Fig. 6, which reports the estimations for both ground and UAV-based estimations.

**Table 1:** Performance of the binary classification to estimate the plant and soil coverage.

Class	Training set	Test set	Accuracies
Plant	1000	2400	99.26% (0.01)
Soil	1000	2400	99.23% (0.02)
OA (%)			99.25% (0.01)
k			0.98 (0.01)

### Geophysical data analysis

**Figure 7:**
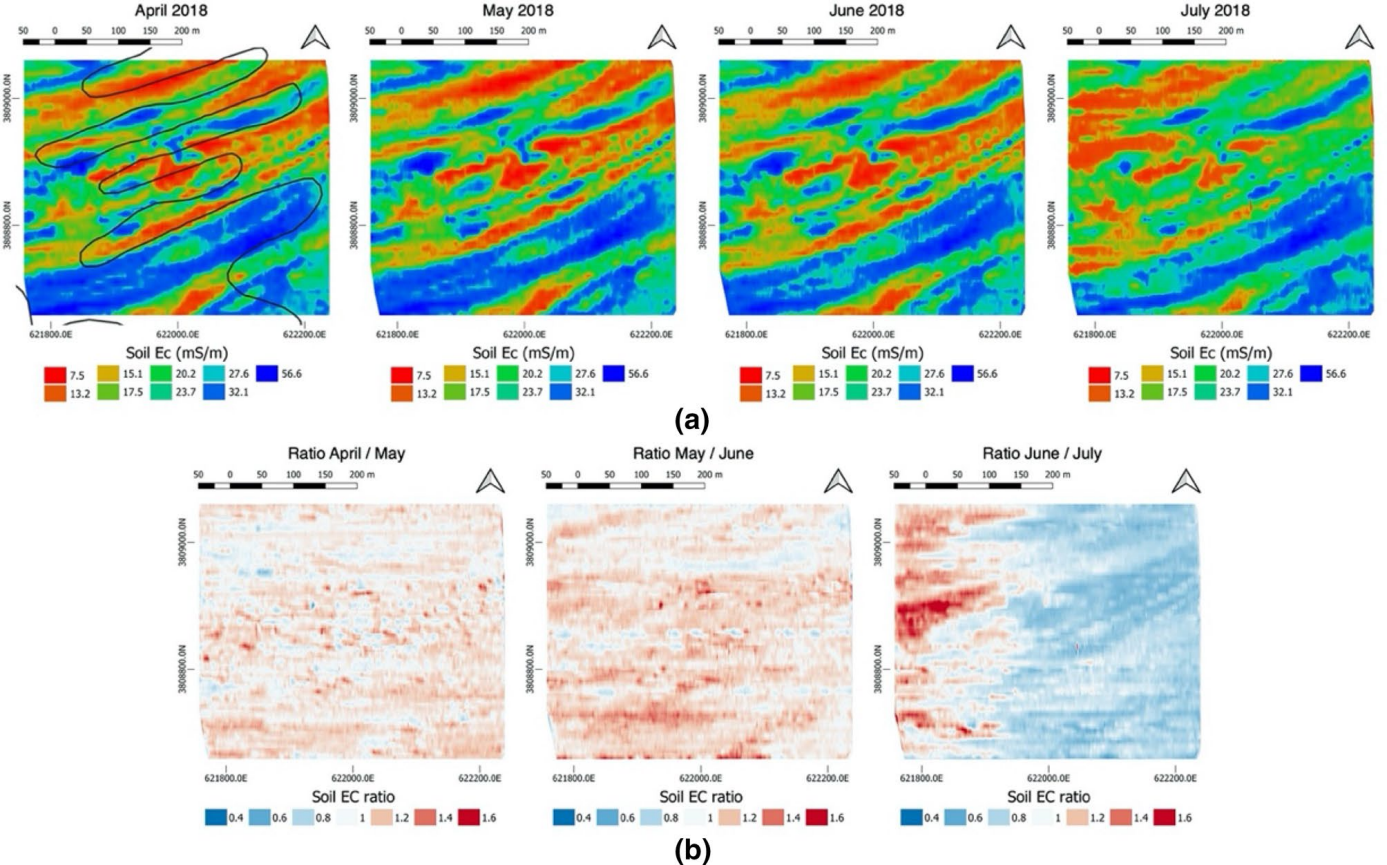
(**a**) Time-series of soil EC_a_ acquired during the growing season. (**b**) Maps of the soil EC_a_ ratio between adjacent data collection. The soil EC_a_ seems consistent between April and June, showing an almost uniform slight decrease (ratio about 1.2). A strong variation can be noticed between June and July, with the west area becoming dryer. The black lines overlaped on the april acquisition represent the gSSurgo boundaries. Maps made with QGIS (v 3.6, https://www.qgis.org)^36^.

Figure 7a shows the interpolated soil EC_a_ data after applying the temperature and spatial corrections for top 1 m of soil. The maps reveal the alternating high and low soil EC_a_ regions in a diagonal pattern, represented by blue and red colours, respectively. This pattern is in agreement with the one shown in the gSSurgo map as well as the soil type division (shown on the april acquisition), with Hebert profile coniciding with high soil EC_a_ areas. The overlap between SSurgo map and our geophysical survey shows a great agreement but also a mismatch between the soil type boundaries. The soil EC_a_ ratio maps (Fig. 7b) computed between consecutiver acquisitions provide a visual interpretation of the possible temporal-spatial variability of soil properties. From the maps, we can see that soil EC_a_ is consistent from April through June (Fig. 7b, left and center), with the ratio maps showing a slightly general decrease in soil EC_a_. A large shift in soil EC_a_ is visible in the west area in the June–July ratio map (Fig. 7b, right). The increase in the ratio (shown in red colour) indicates a decrease in the soil EC_a_ measured in July, due to a possible decrease of soil moisture. Such a decrease is confirmed by the in situ soil-moisture sensors in the east and west regions (Supplementary Figure S2), showing that the irrigation events are well recorded by sensors located at the east side (A1 and A2), whereas the irrigation at the west side (D1 and D2) seems less effective for both shallower and deeper sensors.

**Figure 8:**
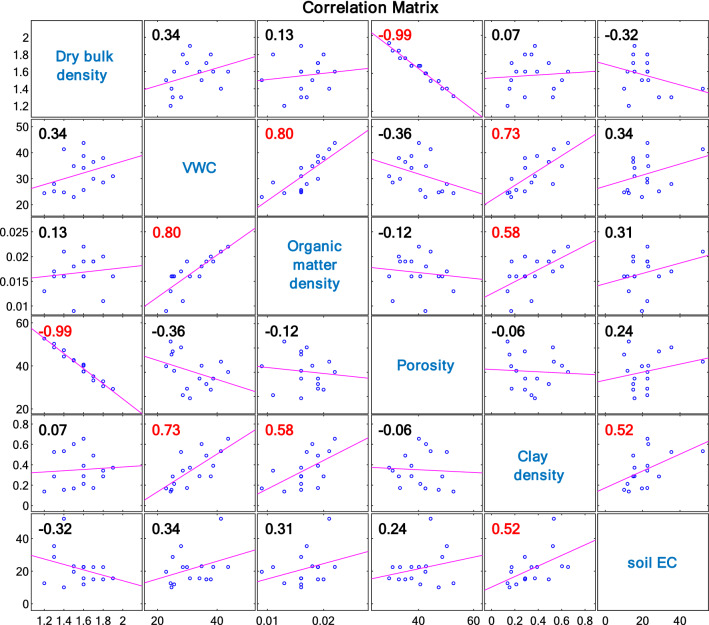
Correlation matrix between soil EC_a_ and soil properties: dry bulk density (g/cm^3^), volumetric water content (VWC) (%), organic matter density (g/cm^3^), porosity (%), and clay density (g/cm^3^). Values that are significant are shown in red (alpha = 0.05).

To better understand the soil EC_a_ data, we performed a statistical analysis between the soil EC_a_ collected in April and the soil properties derived by the analysis of the soil samples, such as clay density (g/cm^3^), porosity (%), dry bulk density (g/cm^3^), volumetric water content (VWC) (%), and organic matter density (g/cm^3^). The resulting correlation matrix (Fig. 8) shows that soil EC_a_ correlates mainly with clay density, which can be seen as an indicator of water retention and of soil with poor drainage properties, as clay density correlates with VWC.

### Co-variability between plant characteristics, soil EC_a_, and yield

**Figure 9:**
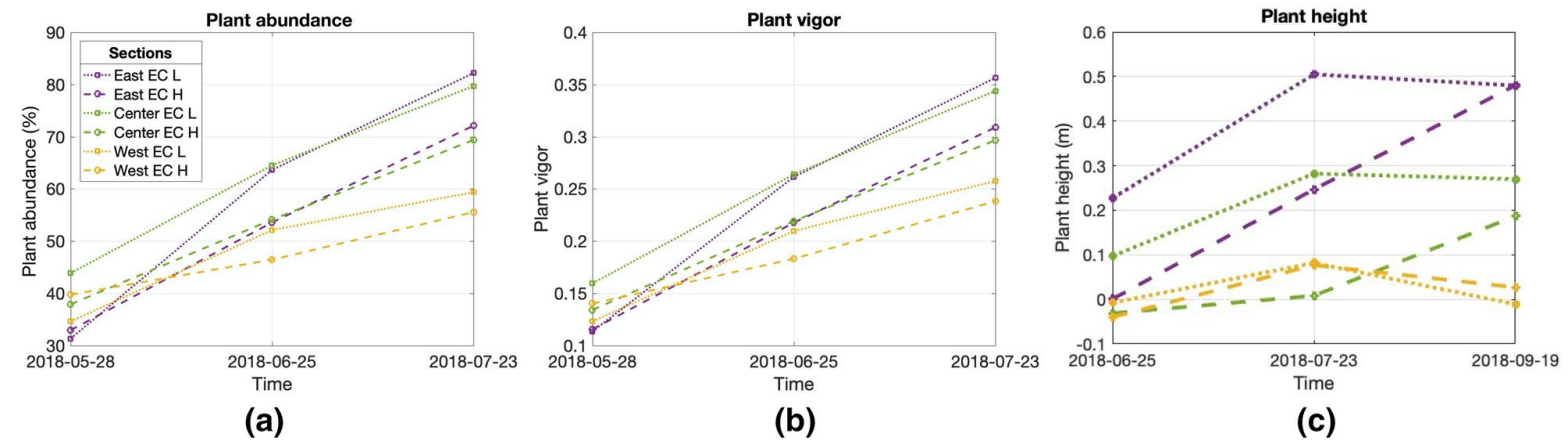
Run charts for time series of plant characteristics: (**a**) plant spatial abundance; (**b**) plant-specific vigor computed in terms of GCC index; (**c**) plant height. The data are reported as average values for each of the three sections (east, center, west, idnetified by different colors), considering high and low values of soil EC_a_.

The results from the analysis of UAV-products showed heterogeneous spatial patterns in terms of plant development. This can be seen in the time series collected during the growing season, shown in Fig. 6, in which plant spatial abundance, plant-specific vigor, and plant height vary drastically within the crop. Such heterogeneity is investigated in more detail in Fig. 9, which reports the analysis of the time-series collected during the growing season. To capture the topographic gradient, the field was divided into three section, east (higher in elevation and close to the water pump), center, and west (lowest in elevation and far from the water pump) (Fig. 1). Such a division has been commonly used in hillslope hydrology and biogeochemistry studies, which focuses on water flow, soil moisture, and associated biogechemical processes^57, 58^. Alternative methodlogies, such as clustering, can be used to identify regions that shows specific temporal-spatial soil–plant dynamics^59^. Within each section, we capture the heterogeneity in soil texture by identifying areas of high a low soil EC_a_ according to the acquired geophysical data and quantified the impact on plant development by comparing plant properties within this areas. Here, we used the soil EC_a_ data acquired in April, as it is not affected by management activities, such as irrigation, and the signal would be driven mainly by soil texture.

The results of the analysis indicated a general decline of plant vigor, spatial abundance, and height from east to west. While plant vigor and spatial abundance consistently increase for the east and central areas from May to July, these plant properties only slightly increase for some of the locations in the west section, and even decline for others in the west. Such a decline can be seen as early as June. Each section is characterized by a different temporal behaviour, with plant spatial abundance and vigor strongly correlated. The highest values of plant spatial abundance, plant-specific vigor, and plant height are observed in low EC_a_ areas within the east section, whereas the west section corresponds to the lowest growth rate. In west seaction, where the plant performance are much lower compared to both the east and canter areas of the field, the relationship soil EC_a_/plant performance is inverted, with higher plant spatial abundance and vigor coinciding with high soil EC_a_.

**Figure 10:**
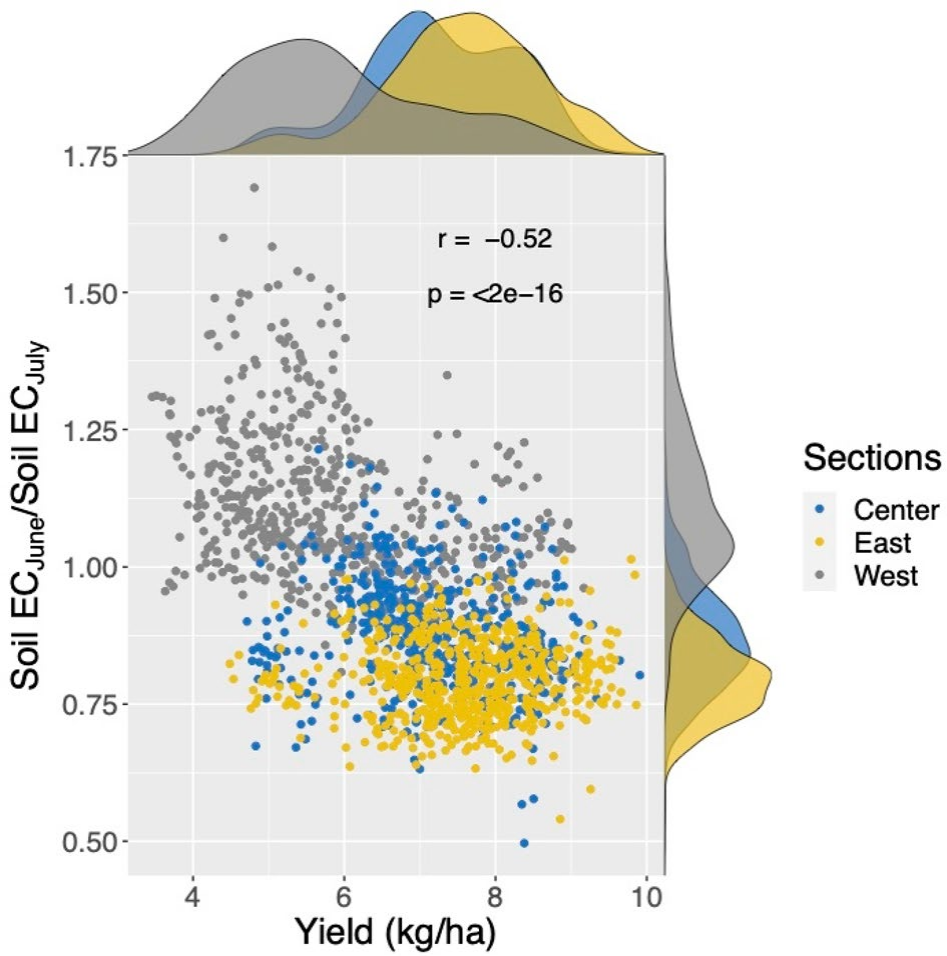
The negative correlation between crop yield (kg/ha) and the ratio of soil ECa of June and July shows the decrease in crop yield corresponding to the decrease in soil moisture in the west area.

We investigated the relationship between the temporal variability of soil EC_a_ computed as ratio (Fig. 7b), and yield (Fig. 3). The soil EC_a_ ratio is used here to account for a possible decrease in soil moisture. The result of this comparison, depicted in Fig. 10, shows a quite different density distribution in yield (lower values) in the west area compared to the east and center areas. The negative correlation between crop yield and the ratio of soil EC_a_ of June and July indicates the decrease of soil moisture is linked to the decrease in the yield compared to the east and center areas. Such conditions produced a yield of one-third less than the one of the previous growing season (Supplementary Figure S3).

We further investigated the correlations between soil EC_a_ and the various plant properties by evaluating the Pearson’s correlations (r) and the corrected Pearson’s correlation (corr) based on Cliffor’d methods . The results of the analysis (Table 2) indicate a general negative correlation between soil EC_a_ and the plant spatial abundance, the plant vigor, and the plant height such that lower soil EC_a_ is associated with higher plant spatial abundance, vigor and height. During the growing season, the negative correlation becomes more pronounced during the summer period, particularly in the east and center sections. There are differences among east, center, and west sections. The negative correlation is smaller in the west section. The negative correlation is consistent in the center and east sections. In the western section, the magnitude of the correlation drops between June and July, implying a major shift has occurred. The results from the Clifford’s method, which consider the spatial association between spatial datasets, showed that plant height is not significantly correlated with soil EC in the east areas. We also investigated the correlation between elevation and soil EC. The results of the analysis showed a non-sinigificat correlation between elevation and soil EC, when spatial association between the two datasets is taken into consideration.

**Table 2:** Pearson’s (r) and corrected Pearson’s (corr) correlation coefficients computed between the average soil EC_a_, plant properties, and elevation. The table reports the correlations computed for the east, center, and west areas. Statistical significance is given by *p < .05, **p < .01, ***p < .001, (−) for non significance.

	Date	West		Center		East	
		r	Corr	r	corr	r	corr
Soil EC versus plant spatial abundance	2018–05-28	0.23***	− 0.09 (−)	− 0.36***	− 0.41***	− 0.03 (−)	− 0.03 (−)
	2018–06-25	− 0.22***	− 0.35***	− 0.56***	− 0.66***	− 0.52***	− 0.57***
	2018–07-23	− 0.01 (−)	− 0.1 (−)	− 0.51***	− 0.57***	− 0.49***	− 0.48**
Soil EC versus plant vigor	2018–05-28	0.21***	0.07***	− 0.40***	− 0.45***	− 0.02 (−)	− 0.08 (−)
	2018–06-25	− 0.24***	− 0.37***	− 0.57***	− 0.66***	− 0.53***	− 0.58***
	2018–07-23	− 0.03***	− 0.13***	− 0.54***	− 0.59***	− 0.50***	− 0.50**
Soil EC versus plant height	2018–06-25	− 0.20***	− 0.22 (−)	− 0.51***	− 0.56***	− 0.58***	− 0.60 (−)
	2018–07-23	− 0.09*	− 0.11 (−)	− 0.52***	− 0.49***	− 0.53*	− 0.49 (−)
	2018–09-19	− 0.34*	− 0.05 (−)	− 0.29***	− 0.34 (−)	− 0.11*	− 0.10 (−)
Soil EC verss elevation		− 0.49***	− 0.38 (−)	− 0.32***	− 0.32 (−)	− 0.57 (−)	− 0.60 (−)

The results of the analysis of the relationship between yield, plant properties, soil EC_a_, and elevation are reported in Table 3. The results show a general positive correlation between yield, plant spatial abundance, vigor, and height such that the higher plant spatial abundance, vigor and height are associated with higher yield, in particular during the summer period. The corrected correlation confirmed the significance in most of these cases. Elevation resulted not sinigificantly correlated to yield, when spatial associatiation is considered.

**Table 3:** Pearson’s (r) and corrected Pearson’s (corr) coefficients computed between the yield, plant properties, soil EC_a_, and elevation. The table reports the correlations computed for the east, center, and west areas. Statistical significance is given by *p < .05, **p < .01, ***p < .001, (−) for non significance.

	Date	West		Center		East	
		r	Corr	r	Corr	r	Corr
Yield versus Plant spatial abundance	2018–05-28	0.15***	0.14 (−)	0.02 (−)	0.06 (−)	0.15***	0.07 (−)
	2018–06-25	0.53***	0.56**	0.26***	0.28*	0.23***	0.22*
	2018–07-23	0.67***	0.69**	0.38***	0.40**	0.32***	0.30*
Yield versus Plant vigor	2018–05-28	0.17*	0.16 (−)	0.04 (−)	0.07 (−)	0.17*	0.09 (−)
	2018–06-25	0.54***	0.57**	0.26***	0.27**	0.23***	0.22*
	2018–07-23	0.67***	0.69**	0.37***	0.39**	0.32***	0.29*
Yield versus Plant height	2018–06-25	0.09*	0.21**	0.22***	0.20 (−)	0.19***	0.21**
	2018–07-23	0.60***	0.57**	0.42*	0.46**	0.46***	0.40**
	2018–09-19	0.53***	0.41**	0.32*	0.37**	0.14*	0.17 (−)
Yield versus Soil EC		0.12*	0.06**	− 0.24***	− 0.21 (−)	− 0.11**	− 0.11**
Yield versus elevation		− 0.14*	− 0.03 (−)	0.02 (−)	0.01 (−)	0.16*	0.14 (−)

## Discussion

This study explored the integration of remote sensing and geophysics to investigate the above/below-ground or soil–plant dynamics that influence plant growth and harvest yield in an agricultural ecosystem. To capture the plant dynamics, we proposed a novel UAV data processing pipeline to estimate plant spatial abundance and plant-specific vigor using high-resolution images (10 cm per pixel). We captured the soil texture and soil moisture variability using surface EMI surveys. Although both technologies—UAV and EM—have been used extensively in agriculture, the major contribution of this study is the integration of these datasets to enhance our understanding of the spatiotemporal variability of soil–plant interactions and to identify the main drivers on crop yield.

In this work, we use an RGB camera to extract plant characteristics, such as plant spatial abundance, plant height, and plant-specific vigor, as well as topographic properties. Plant spatial abundance was computed as the area occupied by the plants within a grid unit, where plants were detected by using a machine-learning based classification approach that exploits spectral and spatial features infromaton. By applying this method to sub-meter resolution UAV images, we were able to estimate plant spatial abundance since the early stage. Such information is critical to identify anomalous areas, where the plant development is affected. Because in early stage, plants are spatially separated, the resulting abundance map can be used to provide an estimate of the sprout density. The plant-specific vigor was computed considering only pixels associated with plants extracted during the plant spatial abundance estimation. This allowed us to minimize the effect of the soil when averaging into the grid unit and preserve plant-specific greenness information.

Although the plant spatial abundance and plant-specific vigor are expected to provide independent information, they are highly correlated. One reason is that high plant spatial abundance areas are expected to have plants with higher biomass, and bigger leaves, which also influences the spectral signal and the computation of the GCC. Additionally, low plant spatial abundance areas are characterized by plants with few or smaller leaves and possibly with more woody parts exposed, which again would decrease the values of plant vigor in these areas.

Plant height was the third metric for plant characterization computed from the RGB images, based on structure-from-motion methods and it is a measure of the plant structure. To minimize the estimation error, we use an RTK-GPS system to ensure that UAV acquisitions are co-registered with errors in x, y, and z around the centimeter scale. Plant height had a similar behaviour to plant spatial abundance and plant-specific vigor, although the difference between low/high soil EC_a_ areas in the east and center sections is more pronounced. This would indicate that plant height, which is a proxy for plant structural information, is more sensitive to water stress due to changes in the environment, such as soil moisture content, affecting the physiology of the plant.

The geophysical surveys based on EMI system provided a high-resolution image of the spatially heterogenous soil properties of the field under study. The temperature and spatial shift corrections were necessary to provide a reliable interpretation of the readings. In particular, the temperature correction is critical when investigating the spatial–temporal variability of soil EC_a_ due to variability in soil moisture^52^. Our EMI data was effectively used in our analysis to capture the soil moisture shift in the west section, which caused the changes in the correlations between soil and plant signatures.

The analysis showed that the spatial heterogeneity in plant spatial abundance, plant-specific vigor, and plant height was influenced by soil textural variability (clay content; represented by high/low EC_a_), soil moisture, and topographical gradient (represented by west, center, east sections). In particular, soil EC_a_ was negatively correlated with plant spatial abundance, and plant-specific vigor, indicating that areas with higher clay content (higher EC_a_) have a negative impact on the plant development. Plant height resulted negatively correlated, however resulted not significant within the east and west sections, when spatial association is considered. The general negative correlation between plant properties and soil EC could be attributed to short-term soil water saturation during the irrigation period caused by the irrigation system based on furrow. The saturated soil in clay-rich regions with a slow drainage could particularly limit the soil oxygen uptake, negatively impacting the plant development^39^. Such conditions may not apply to other crops with a deeper root system^33^. It is worth noting that while topographic gradient represents the main driver on water distribution, it is not aligned with either soil EC_a_ measurements and gSSurgo soil map (as shown in Fig. 7a and supported by the statistical analysis in Table 2).

The correlation between soil EC_a_ and plant signatures is consistent over time but heterogeneous; the correlation in the west section decreased in July. Such a shift was due to a change in soil conditions that affected plant development. The change was captured by both the UAV-based products and the geophysical survey. The plant spatial abundance and plant height showed a decrease in performance in the west area during the early maturity period, indicating the interruption of the plant growth. Using the time-series EMI data and applying the ratio between acquisitions, we were able to capture the decrease of soil moisture and its spatial extent, which in turn affected the plant development in that area. The change in soil conditions occurred after June can be attributed to the cumulative results of the few precipitation events during the vegetative stage and to the non-uniform irrigation, for which topographical gradient represented the main control.

Part of our analysis was to understand the link between soil–plant dynamics and crop yield. The negative correlations between yield and soil EC_a_ in the east and center areas showed the impact that soil texture has, not only on the plant development, but also on the final yield. Because of the water limiting scenario in the west area, results reported a positive correlation between areas with higher clay content and crop yield, as these areas have higher water retention capabilities.

The statistical analysis showed that plant properties are significantly correlated with crop yield. Such correlations, however, are low for those data acquired in the early vegetative stage. This is due to the non-uniform plant distribution at emergence^60^ and to the presence of areas with low initial plant spatial abundance but have improved during the growing season. Such improvement depends on soil condition, temperature, as well as being in competition among plants^60^. On the other hand, the early assessment showed several areas identified as potentially anomalous that persisted during the growing season, with correlations increased during the growing seasons, proving the efficacy in performing early assessment. Additionally, early assessment conducted with UAV-based imaging together with photogrammetry techniques, allowed us to derive the topographical gradient, which represented an important driver for water management with strong implications on both plant development and soil–plant relationships. In the late season, we can see a decrease of the correlation and statistical significance between yield and plant height in September (see plant height in September 19th), in particular in the east and center areas. The decrease in correlation is due to the presence of areas in which the soybeans are approaching the maturity stage, when leaves turn yellow to then fall. Also, plants that have grown high tend to fold, causing a general decrease in the UAV-estimated plant height. The UAV acquisition during the September period could potentially be used to retrieve information on the spatial variability of maturity rate, and potentially be used to optimize planting in the next season.

The methodological framework developed in this study—based on time-series analysis of UAV-based images and geophysical data—was able to capture the dynamic plant-soil spatial patterns, and can be easily implemented for precision agriculture as a tool for early assessment and crop monitoring. From our analysis, multitemporal acquisitions are necessary to understand the heterogeneity in soil–plant signatures associated with a crop field. Remote sensing acquisitions should be planned accordingly to the crop growth-schedule to acquire data during critical stages. However, we recognize the difficulty in acquiring a such high-resolution dense data stream. As such, the combination of UAV and satellite imagery could be a good alternative. While the UAV’s high resolution is necessary to perform an early assessment of sprout coverage, providing insight on possible anomalous areas that could be treated timely, multiple satellite acquisitions can provide insight on the possible plant degradation. Particular attention should be given to acquisitions during the late maturity stage, since vegetation indices could provide misleading information due to plants phenology changes during that period. Additionally, an early geophysical survey can guide the ground sampling in order to assess soil physical–chemical properties to optimize amendment applications. Such assessment could be performed once a year prior to planting since soil texture wound not change over time, while a sensor network could be used to track soil moisture variability during the growing season.

While this study focused on a soybean crop, the methodology is general and it can be applied to different crops to provide a better understanding of the underlying causes of a possible spatial variability in plant growth, and provide insights for the next season.

## Conclusion

This study explored the joint use of remote sensing and geophysical data to investigate soil–plant interactions in a soybean crop field. We proposed a novel pipeline to extract informative products, such as plant spatial abundance and plant-specific vigor from multitemporal high-resolution UAV data to capture plant dynamics since the early-stage of the growing season. We exploited an EMI system to measure high-resolution and spatially extended soil properties by measuring soil EC_a_.

The spatial analysis of the multisource data stream showed a strong spatial heterogeneity in soil properties. A similar spatial pattern was captured by plant spatial abundance and plant vigor products already at the early-stage, indicating the strong impact of soil properties on plant development.

In particular, a significant negative correlation between plant properties and soil EC_a_ indicated that increased amount of clay had negative impact on plant development. In addition, our analysis showed that the spatiotemporal variations in soil moisture, imaged by temporal changes in EC_a_ and influenced by topography, impacted the plant development. Thus, the topographic gradient represented a major control on the water management, with implications on the plant development and crop yield.

The results of this study showed that an effective integration of remote sensing, surface electromagnetic geophysics, and point measurements enables a better understanding of the dynamics that characterize soil–plant interactions in agriculture ecosystems. Furthermore, the ability of the developed methodology in characterizing above and below surface properties would provide valuable information to guide crop management from the early season and throughout the entire growing season.

## Supplementary Information

Supplementary Information
