# Supplementary material for: Influence of soil heterogeneity on soybean plant development and crop yield evaluated using time-series of UAV and ground-based geophysical imagery
Source: Sci Rep. 2021 Mar 29;11:7046. doi: 10.1038/s41598-021-86480-z (PMC8007594; doi:10.1038/s41598-021-86480-z)
Supplement: Supplementary file 1 — Supplementary Information [file 41598_2021_86480_MOESM1_ESM.pdf]

Supplementary Information

Influence of Soil Heterogeneity on Soybean Plant Development and Crop Yield Evaluated Using Time-series of UAV and Ground-based Geophysical Imagery

Authors:

[a]Nicola Falco, [a]Haruko M. Wainwright, [a]Craig Ulrich, [a]Florian Soom, [a]Baptiste Dafflon, [a]John Peterson, [b]James Bentley Brown, [c]Karl B. Schaettle, [d]Malcolm Williamson, [d]Jackson D. Cothren, [e]Richard G. Ham, [f]Jay A. McEntire, [a]Susan S. Hubbard.

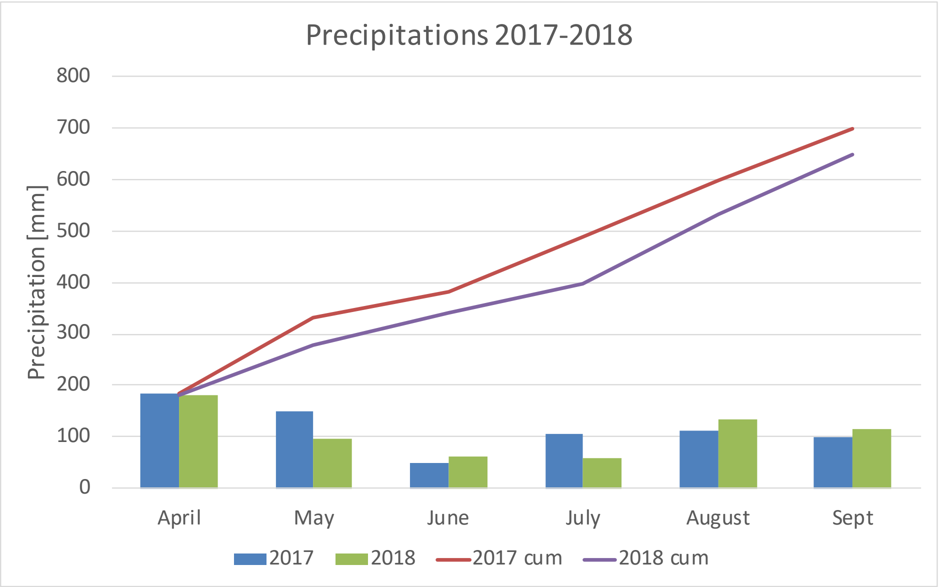

Figure S1: The graph shows monthly and cumulative precipitations for 2017 and 2018. Weather data for 2017 were obtained by a local weather station located in Stuttgart, AR (34.48° N, 91.55° W). Reported cumulative precipitation for the period April – September was about 700mm. Weather data for 2018 were measured by a weather station located adjacent to the study area. Based on the data collected, the 2018 growing season had less precipitations during the vegetative period compared to 2017, with a cumulative precipitation of about 650 mm.

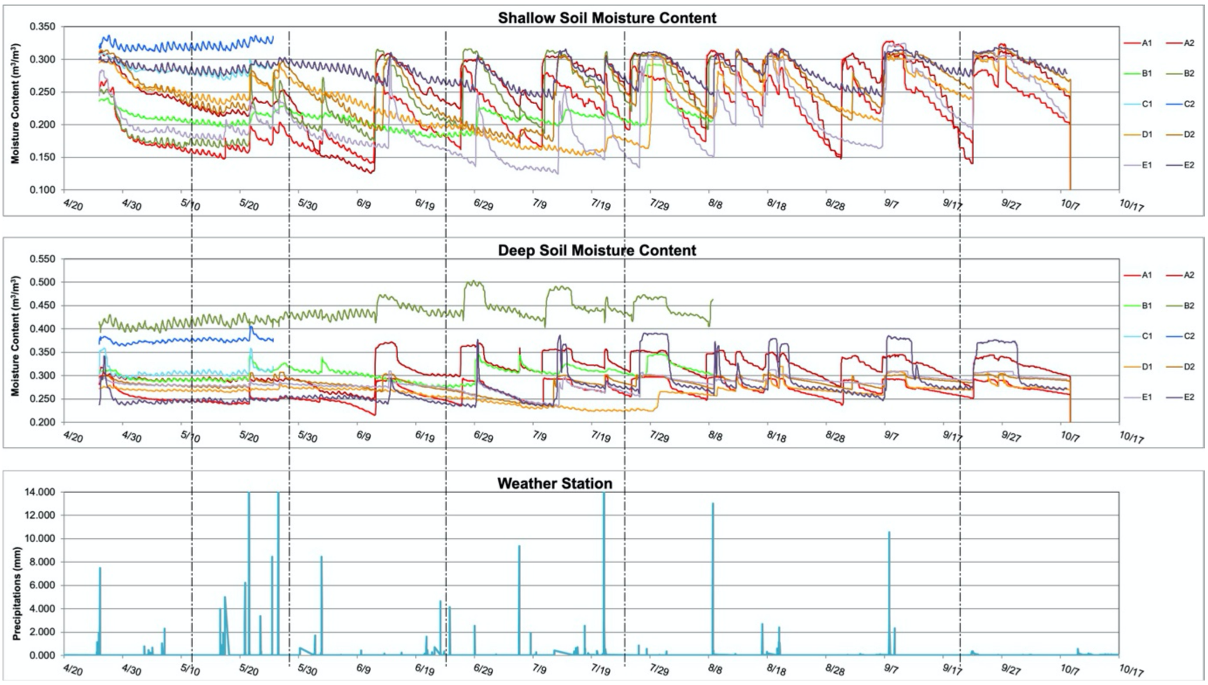

Figure S2: In situ data collected in 2018 by 12 soil sensors located within the field (A, B, C, D, E, Figure 1 of the manuscript). Each location has a north and south side (e.g., A1 and A2). For each side, we placed a shallow (12 cm deep, top graph) and a deep (25 cm, center graph) sensor. Unfortunately, sensors in C stopped working after planting. Sensors in A, B, and D are positioned along the same transects. The bottom graph reports precipitation recorded by the weather station. Black vertical lines indicate the UAV acquisitions. Soil sensors along the transect 1 (A1, B1, D1) show that irrigation events are captured in A1 (both by deep and shallow soil sensors). Sensors in B1 captured mainly rain events, and irrigation events starting at the end of July. Sensors in D1 show a decline in water content starting from May, after the rain events, until mid-July. Along transect 2, we see that sensors in A2 and B2 captured irrigation events, while sensors in D2 have missed events.

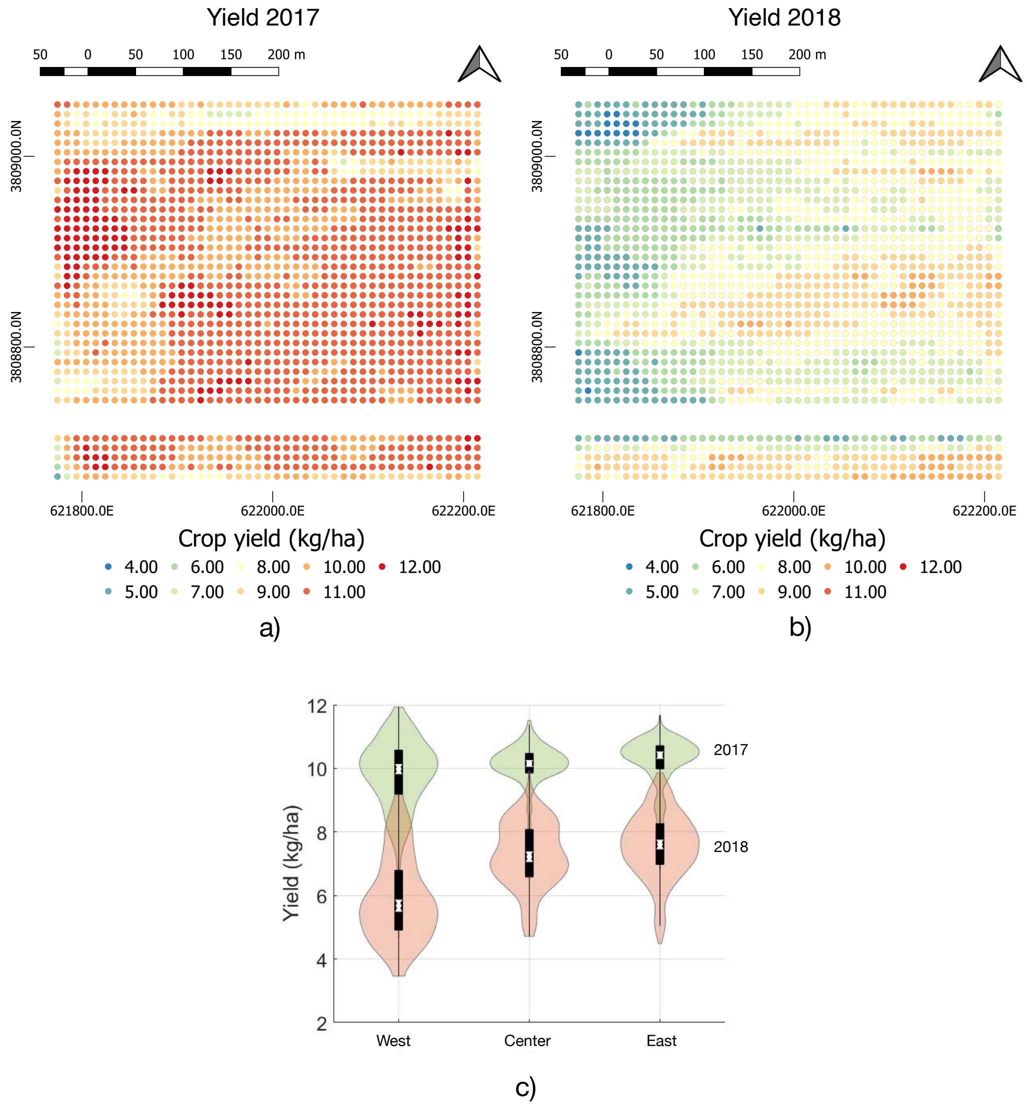

Figure S3: Point-cloud maps of the yield acquired in kg/ha by the combine machine for: a) 2017 and b) 2018 growing seasons. c) Violin plots showing the inter-annual difference between the west, center, and east areas. The comparison shows the portion of yield lost in 2018 compared to the one of 2017. Maps made with QGIS (v 3.6, https://www.qgis.org).
